# Evidence of ascariasis in a Celtic newborn from northern Italy

**DOI:** 10.1590/0074-02760250091

**Published:** 2025-12-15

**Authors:** Ramón López-Gijón, Wolf-Rüdiger Teegen, Zita Laffranchi, Daniele Vitali, Albert Zink, Marco Milella

**Affiliations:** 1 Universidad de Granada, Facultad de Medicina, Laboratorio de Antropología, Granada, Spain; 2 Universidade de Évora, Laboratório Hercules, Évora, Portugal; 3 Ludwig-Maximilians-Universität München, Institut für Vor- und Frühgeschichtliche Archäologie und Provinzialrömische Archäologie, München, Germany; 4 Ludwig-Maximilians-Universität München, ArchaeoBioCenter, München, Germany; 5 Universität Bern, Institut für Rechtsmedizin, Abt Anthropologie, Bern, Switzerland; 6 Bernisches Historisches Museum, Bern, Switzerland; 7 Université de La Bourgogne, Dijon, France; 8 Institute for Mummy Studies, Eurac Research, Bolzano, Italy; 9 Università di Pisa, Dipartimento di Biologia, Pisa, Italy; 10 University of Coimbra, Research Centre for Anthropology and Health, Department of Life Sciences, Coimbra, Portugal

**Keywords:** Ascaris lumbricoides, Iron Age, Italy, neonate, palaeoparasitology

## Abstract

**BACKGROUND:**

Infections with *Ascaris lumbricoides* can be traced back to the late Pleistocene by palaeoparasitological analysis. Even today, *Ascaris* infections are still very common worldwide.

**OBJECTIVES:**

In a pilot study, soil samples from the pelvic area of ten individuals from the Celtic necropolis of Povegliano Veronese (northern Italy) were examined using palaeoparasitological methods. The burials date from the 3rd to 1st century Before the Common Era (BCE).

**METHODS:**

The palaeoparasitological methods already proven in earlier studies were applied.

**FINDINGS:**

Positive evidence of *Ascaris* eggs was obtained in three individuals, including a newborn. This neonate is the focus of the article. The causes of a possible *Ascaris* infection in a newborn are discussed.

**MAIN CONCLUSIONS:**

It may represent the oldest documented instance of ascariasis in a neonatal individual.

The roundworm *Ascaris lumbricoides* has a long history of infecting humans, with evidence dating back to the Pleistocene (BP), at least 25,000 years BP.^1^ Despite significant medical advancements, the prevalence of this infection remains alarmingly high in many regions of the world, particularly in the Global South. Moreover, due to increasing global mobility and migration, the once-forgotten disease ascariasis is re-emerging in Europe and North America.^2^

It is estimated that approximately 25% of the world’s population is affected by soil-transmitted helminths (STH),^3^ with nearly one billion individuals infected by *A. lumbricoides* alone.^4^ Alongside dental caries, ascariasis is considered one of the most prevalent infectious diseases globally.

Soil-transmitted helminths include not only the roundworm *A. lumbricoides* but also the whipworm *Trichuris trichiura* and the hookworms *Ancylostoma duodenale* and *Necator americanus*.^3^ ^,^ ^4^

## SUBJECTS AND METHODS

The late Celtic Gallo Roman necropolis at Località Ortaia in Povegliano Veronese (northern Italy) was excavated between 2007 and 2009 by an international team from Italy, Hungary and Germany. The excavation revealed a total of 112 inhumations and 36 cremations, dated to the 3rd to early 1st century Before the Common Era (BCE).^5^ During the cleaning process of the bones, soil samples were routinely collected by WRT from the visceral surfaces of the iliac and sacral bones for further analysis and from the skull area for controls. The skeletal remains were subsequently studied by WRT from anthropological and palaeopathological perspectives.^6^

In 2022, samples from all inhumations were analysed for stable isotopes. Additionally, the pars petrosa of the temporal bone was sampled from a subset of individuals, selected based on their funerary and/or anthropological features, for palaeogenetic analysis.

A pilot palaeoparasitological investigation was conducted by RLG on soil samples taken from the pelvic cavities of ten individuals from Povegliano Veronese-Ortaia. Previous studies have demonstrated that parasitic worm eggs can be preserved in soil from the intestinal areas of humans and animals.^7^ ^,^ ^8^ The rehydration, homogenisation, and micro sieving (RHM) technique^9^ was utilised for the palaeoparasitological study. Bright field transmitted light optical microscopy was used to visualise the samples under 100×, 400×, and 600× magnification, capturing photographs at 600× magnification.^10^ ^,^ ^11^

## RESULTS

Burial 154 contains the well-preserved remains of a neonatal individual, aged 0-3 months based on long bone measurements and dental development. Although sex estimation in immature remains is inherently challenging, the mandibular morphology suggests a female individual.^12^

**Fig. 1:**
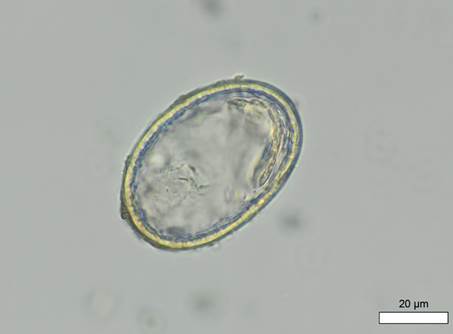
Povegliano Veronese, Loc. Ortaia (Italy), burial 154. Possible female (F > M), 0-3 months. Microscopic image of one of the seven *Ascaris lumbricoides* decorticated eggs associated with the individual (measurements: 58.8 × 40.39 μm). Photo: R. López-Gijón.

**Fig. 2:**
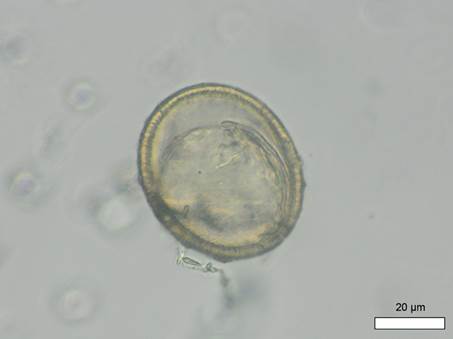
Povegliano Veronese, Loc. Ortaia (Italy), burial 154. Possible female (F > M), 0-3 months. Microscopic image of another one of the seven *Ascaris lumbricoides* decorticated eggs associated with the individual (measurements: 59.08 × 48.51 μm). Photo: R. López-Gijón.

Pathological examination revealed several alterations: new bone formation above the vessel impressions on both parietal bones, slight new bone formation around foramina in the right orbit (the left orbit was not preserved), and extended layers of new bone formation on the long bones. These morphological changes are consistent with a probable diagnosis of scurvy (Morbus Möller-Barlow), indicating an active disease process at the time of death. Palaeoparasitological analysis identified *A. lumbricoides* eggs in three out of ten soil samples analysed, including those from burial 154. In this individual, a total of seven decorticated *A. lumbricoides* eggs were recovered (Figs 1-2). A control sample from the skull area remained negative. The shape and size of the eggs (ovoid/elliptic and ranging between 52-63 μm in length and between 36.1-49.9 μm in width) are compatible with *A. lumbricoides*. As mentioned, the decortication of the outer layer and loss of the characteristic mamillated coat can be ascribed to taphonomic processes typical of ancient specimens. The fact that all recovered *Ascaris* eggs were decorticated suggests the influence of taphonomic processes which are likely also responsible for the small number of identified eggs in the samples. In the neighbouring Lombard burial ground of Povegliano Veronese, no parasitic eggs were detected in the 14 soil samples examined.^13^

## DISCUSSION

*Ascaris lumbricoides* requires approximately 9-11 weeks to develop from infection (via the ingestion of fertilised eggs) to sexually mature individuals.^4^ Case studies have demonstrated that human neonates can be infected with *A. lumbricoides*.^14^ ^,^ ^15^

One such case, published by Chu et al., involved a two-day-old infant delivered via Caesarean section.^14^ A living female worm was recovered from the infant’s faeces, alongside fertilised *Ascaris* eggs found in the new-born’s faeces, amniotic fluid, and the mother’s stool. The authors proposed an intrauterine transmission, suggesting that larvae may have crossed the placenta, entered the umbilical vein and foetal circulation, and developed into adult worms in utero. Adult female worms were also discovered in the placenta.^14^

Another case, reported by Rathi et al., described a 45-day-old infant suffering from intestinal obstruction caused by *Ascaris* infection.^15^ The excretion of macerated worms precluded determining whether the worms were sexually mature or in a pre-patent stage. Possible sources of infection included honey or contaminated water administered in the first days of life, though a transplacental transmission route could not be ruled out.

Further evidence of congenital transmission via the placenta is suggested in a case of neonatal ascariasis reported by Da Costa-Macedo and Rey.^16^ This route of transmission is well-documented in *Toxocara canis*, another member of the Ascaridae family.^16^ Neonatal *Ascaris* infections are often associated with nutritional deficiencies, including lactose digestion issues, which can severely impair weight gain in neonates.^17^ For example, the neonate described by Chu et al., born at eight lunar months, weighed only 2010 g with a body length of 45 cm.^14^

In our case, precise age at death could not be determined beyond the range of 0-3 months due to inherent limitations in anthropological age estimation. Thus, the observed parasitic infection may have been congenital or acquired in the first days of life. The exact cause of death for the neonate remains undetermined. While intracranial bleeding may have been a contributing factor, we cannot exclude intestinal obstruction due to *Ascaris* infection.

The case of Povegliano contributes to the limited corpus of palaeoparasitological data available for the Iron Age in continental Europe, which is primarily represented by individual coprolites from mining sites (*e.g.*, the salt mines of Hallstatt and Hallein), intestinal samples from bog bodies (*e.g.*, the Döbritz girl and Lindow Man), and the famous early La Tène princely grave of Lavau (France) or communal samples from ditches (*e.g.*, in the Celtic settlement Basel-Gasfabrik).^18^ ^,^ ^19^ ^,^ ^20^ These findings indicate that helminth infections were already widespread among Iron Age communities, occurring across diverse ecological, social, and cultural settings.

In conclusion, although *Ascaris* infections are rare in neonatal individuals, several clinical cases of neonatal ascariasis have been documented in modern contexts. However, to date, this parasite has not been identified in neonates from archaeological contexts.

In the present case, we report what may represent the oldest documented instance of ascariasis in a neonatal individual. The infection could have been acquired via intrauterine transmission or within the first weeks of life. This finding contributes to our understanding of the long-standing relationship between humans and parasitic infections, even among the most vulnerable age groups in ancient populations. A parallel study currently under review, based on additional samples from Povegliano and the nearby necropolis of Seminario Vescovile (López-Gijón et al., Unpublished data), will help better contextualise the case presented in this work.
